# Pulsed Laser Ablation-Induced Green Synthesis of TiO_2_ Nanoparticles and Application of Novel Small Angle X-Ray Scattering Technique for Nanoparticle Size and Size Distribution Analysis

**DOI:** 10.1186/s11671-016-1608-1

**Published:** 2016-10-05

**Authors:** Amandeep Singh, Jorma Vihinen, Erkka Frankberg, Leo Hyvärinen, Mari Honkanen, Erkki Levänen

**Affiliations:** 1 Department of Materials Science, Tampere University of Technology, P. O. Box 589, FIN 33101 Tampere, Finland; 2 Department of Mechanical Engineering and Industrial Systems, Tampere University of Technology, P. O. Box 589, FIN 33101 Tampere, Finland

**Keywords:** Nanoparticles, Pulsed laser ablation in liquids, Nanoparticle synthesis, Nanoparticle size and size distribution analysis, TEM, SAXS, XRD

## Abstract

This paper aims to introduce small angle X-ray scattering (SAXS) as a promising technique for measuring size and size distribution of TiO_2_ nanoparticles. In this manuscript, pulsed laser ablation in liquids (PLAL) has been demonstrated as a quick and simple technique for synthesizing TiO_2_ nanoparticles directly into deionized water as a suspension from titanium targets. Spherical TiO_2_ nanoparticles with diameters in the range 4–35 nm were observed with transmission electron microscopy (TEM). X-ray diffraction (XRD) showed highly crystalline nanoparticles that comprised of two main photoactive phases of TiO_2_: anatase and rutile. However, presence of minor amounts of brookite was also reported. The traditional methods for nanoparticle size and size distribution analysis such as electron microscopy-based methods are time-consuming. In this study, we have proposed and validated SAXS as a promising method for characterization of laser-ablated TiO_2_ nanoparticles for their size and size distribution by comparing SAXS- and TEM-measured nanoparticle size and size distribution. SAXS- and TEM-measured size distributions closely followed each other for each sample, and size distributions in both showed maxima at the same nanoparticle size. The SAXS-measured nanoparticle diameters were slightly larger than the respective diameters measured by TEM. This was because SAXS measures an agglomerate consisting of several particles as one big particle which slightly increased the mean diameter. TEM- and SAXS-measured mean diameters when plotted together showed similar trend in the variation in the size as the laser power was changed which along with extremely similar size distributions for TEM and SAXS validated the application of SAXS for size distribution measurement of the synthesized TiO_2_ nanoparticles.

## Background

Nanotechnology has eminently transformed the technology sector in the last few decades. It includes the atomic level analysis and manipulation of materials. It has been the center of interest for material scientists, chemists, and physicists from the past several decades. The production of nanoparticles accounts for a substantial portion in the field of nanotechnology. This includes production and processing of nanoparticles of several materials such as metals, semiconductors, carbon, metal oxides, and metal carbides.

The prevailing methods for the production of nanomaterials such as graphene are chemical vapor deposition and chemical exfoliation that are toxic and batch-type processes [1]. As these processes use toxic chemicals, therefore, they are precarious and potentially detrimental for the environment. Ogale et al. in 1992 discovered formation of nanoparticles when materials immersed in water were ablated by a pulsed laser [2]. Pulsed laser ablation in liquids (PLAL) allows production of nanoparticles with no by-products [3]. In this method, irradiation of a target material with ultra-short laser pulse leads to formation of high-temperature plasma and removal of material, which has been termed as pulsed laser ablation [4]. Researchers have reported synthesis of nanoparticles of pure metal, metal oxide, and metal carbide by pulsed laser ablation [5–11]. With this clean and versatile technique, high-purity nanoparticle can be synthesized, which are well suited for functionalization [12–14]. The determination of the size and size distribution of nanoparticles, as part of their characterization, is of utmost importance in order to effectively use nanoparticles. The control of nanoparticle size is indispensable; however, in order to control it, it is necessary to quantify the size. The size of nanoparticles can be analyzed by various methods such as dynamic light scattering (DLS), atomic force microscopy (AFM), transmission electron microscopy (TEM), scanning electron microscopy (SEM), differential mobility analysis (DMA), and small angle X-ray scattering (SAXS) [15]. SAXS has several advantages over the conventional methods that are used to analyze the nanoparticles. The list types of samples that can be analyzed by SAXS are extensive, and the sample preparation is very quick especially when compared to preparation for electron microscopy samples. Another advantage of the technique is that the measurements are more reliable since the analysis is made over a much larger number of particles than what can be measured from TEM images [15]. DLS and DMA are not able to evaluate particles in thin films while it is possible with SAXS. DLS measurement relies on the temperature and concentration of the solution [16]. Agbabiaka et al. have proposed small angle X-ray scattering as a promising method for measurement of nanoparticle size [15]. Vippola et al. have reported SAXS as an excellent method for size analysis of small spherical particles [17]. However, this technique has never been applied and verified for pulsed laser-ablated TiO_2_ nanoparticles to the best of our knowledge. So, the aim is synthesis of TiO_2_ nanoparticles followed by application and verification of small angle X-ray scattering for characterization of TiO_2_ nanoparticle size.

The present study deals with (i) synthesis of TiO_2_ nanoparticles by PLAL, (ii) characterization of TiO_2_ nanoparticles, and (iii) application and verification of SAXS for size and size distribution analysis of TiO_2_ nanoparticles.

## Methods

**Fig. 1:**
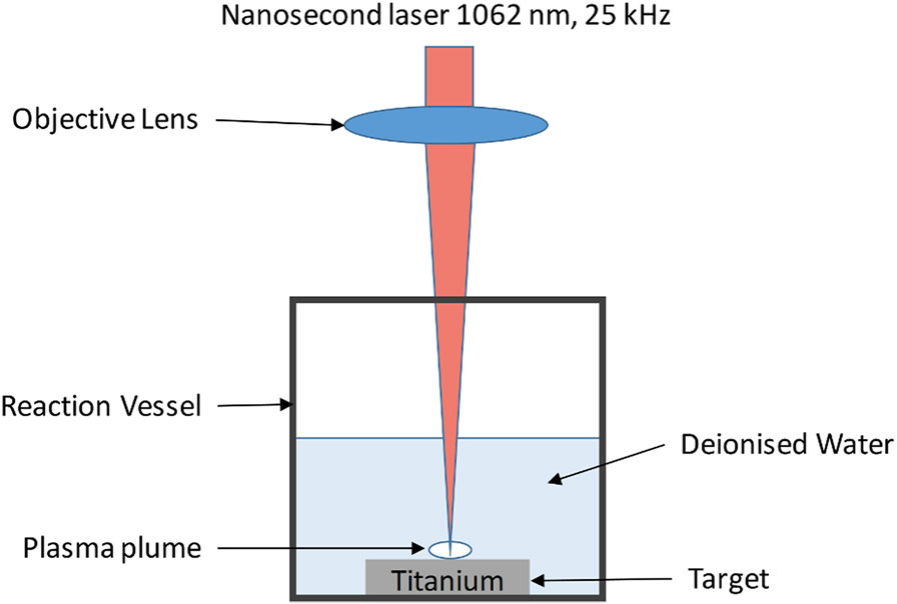
Schematic for pulsed laser ablation in liquid

For carrying out the pulsed laser ablation test, the experimental setup (schematic shown in Fig. 1) consisted of a nanosecond 85W fiber laser (1062 nm, pulse length 500 ns, 25 kHz), titanium target, test vessel, and a computer-controlled XY scanner connected to the laser to scan it on a predetermined area on the target. The target, immersed in deionized water (DIW), was irradiated with this laser from the top, perpendicular to the plane of the target. The laser beam was focused on the target using a lens having focal length 160 mm. The maximum laser fluence possible with this laser was 58.92 J/cm^2^. As the spot diameter of the laser varied with the laser power, the measured laser fluences of the laser also varied from 34.65 J/cm^2^ at 20 % laser power (LP) to 58.92 J/cm^2^ at 40 % LP and to 43.22 J/cm^2^ at 50 % LP. The ablation threshold fluence for titanium was reported to be ~4.5 J/cm^2^ with an Nd:YAG laser (1064 nm, pulse length 4.5 ns, 10 kHz) [18].

### Materials and Synthesis

Titanium (99.99 % pure), obtained from Goodfellow Cambridge Limited, in the form of foil of thickness 3.2 mm, length 50 mm, and width 50 mm was cut into several smaller targets by using Struers Accutom-50 precision cutter. All these targets were identical in size, 15 mm × 15 mm, and ablated area on target surface was 8 mm × 8 mm for each target in ablation experiments. For the experiments, only DIW was used and no chemicals were used for the nanoparticle synthesis. Small LDPE bottles, ordered from VWR International Limited, were used for storing the synthesized nanoparticle suspensions. The ultrasonically cleaned target was fixed inside the test vessel at its base and then filled with DIW such that the thickness of the water film above the target was 5 mm. For each ablation experiment, the scanning area or ablated area was 8 mm × 8 mm and the scanning speed was 2000 mm/s. The scanning parameters were the same for each target in all ablation experiments. The direction of scanning was top to bottom followed by right to left which was repeated loop by loop of the scanning cycles. Each scanning loop was 2.566 s long. The number of loops was 720 corresponding to an ablation test of 30-min duration. This high value of scanning speed was chosen in order to have the least number of laser pulses coincident at a point.

### Characterization of Synthesized Particles

TEM imaging was performed with a Jeol JEM-2010 microscope. For imaging the nanoparticles, the acceleration voltage for TEM was 200 kV in each case. The TEM samples were prepared from freshly ablated suspensions so that there is least agglomeration. Nanoparticle dispersions were dropped on copper grids with a holey carbon film. From the same samples, electron diffraction patterns were also obtained. These techniques were used to characterize the shape, size, and crystallinity of nanoparticles. X-ray diffraction and X-ray scattering measurements were performed on a Panalytical Empyrean Multipurpose Diffractometer with anode material copper. It used Cu Kα radiation (wavelength 0.15418 nm) powered by X-ray generator at 45 kV and 40 mA and a solid-state pixel detector, PIXcel3D, which measured the scattered intensities as a function of scattering angle (2*θ*). For X-ray diffraction (XRD) measurements, the scan range was 20.00°-80.95° with a 0.05° step size. It used a soller slit with 0.04 radian opening. For SAXS measurements, the scan range was from −0.12° to 5.01° with a 0.01° step size and it utilized a parallel beam X-ray mirror for Cu radiation. SAXS was performed on nanoparticle suspensions while for XRD measurements, the nanoparticle suspensions were dried to obtain nanoparticle powder. For SAXS sample, equal volume of each suspension was enclosed between two Mylar foils (which were X-ray transparent) and finally placed between the respective circular transmission holders for each SAXS sample. DIW enclosed between two Mylar foils was used as the background sample. The phase identification of the peaks in the XRD pattern was done with Panalytical HighScore Plus software (version 3.0.5).

### Measurement of Nanoparticle Size and Size Distribution

The nanoparticle sizes were measured by SAXS and compared with the nanoparticle size measurements from TEM image analysis done manually. With SAXS measurement, we obtained the mean particle size, most frequent particle size, and the size distribution of particles by volume. The experimental data from SAXS was analyzed by Panalytical’s EasySAXS software (version 2.0a) to obtain the volume-weighted particle size distribution, D_v_, by indirect Fourier transform method. With this method, the result from EasySAXS is a plot between particle size and distribution volume ratio, i.e., D_v_(R), showing size distribution of nanoparticles by volume. In the second method used, the diameters of 100 nanoparticles were measured from TEM images. This method was time-consuming. After measuring the diameters, the data was represented in the form of a histogram in order to analyze the size distribution. Mean diameter of the nanoparticle was calculated from 100 measured diameters. Finally, in order to observe the pattern and compare both the measuring techniques, the histogram from TEM image analysis was plotted along with size distribution curve obtained from SAXS measurement. The samples for SAXS were prepared several hours after ablation while TEM samples were prepared from freshly ablated suspensions. In order to know the crystallite size, peak analysis was performed on the peaks in the XRD pattern by using Panalytical HighScore Plus software (version 3.0.5) which uses line profile analysis algorithm based on the Scherrer equation for determination of crystallite size.

## Results and Discussion

The process of PLAL can be divided into five stages: (i) irradiation of target with pulsed laser to form plasma, (ii) relaxation of plasma and formation of a cavitation bubble, (iii) nucleation and growth of nanoparticle in the cavitation bubble, (iv) collapse of the cavitation bubble to release nanoparticles in surrounding liquid, and (v) irradiation of synthesized nanoparticles by laser pulses. With PLAL, the nanoparticles are formed by two distinct ablation phenomena, one is with the jet-shaped shadow that ejects solid fragments and droplets from the surface and the second is the formation and growth of nanoparticles from ejected target atoms inside the cavitation bubble [19]. With a microsecond laser pulses, the material removal from the target surface is predominantly by vaporization and boiling while with nanosecond and picosecond laser pulses, there is a coexistence of both direct photoionization and thermal ablation mechanisms, such as vaporization and boiling [20]. The generation and growth of nanoparticles occurs inside the cavitation bubbles, where the ejected atoms, clusters, and droplets collide and aggregate to form nanoparticles [19, 21]. This is followed by a collapse of the cavitation bubble to release the nanoparticles into the surrounding liquid [21]. This collapse of the cavitation bubble occurred, for instance, 610 μs after irradiation in a study by Lazic et al. when they used a 9.5 ns, 1064 nm Nd:YAG laser [22].

Titanium is chemically active and reacts strongly with the vapors of surrounding liquid resulting in formation of titanium compounds, and in this regard, PLAL is a chemical bottom-up process. During PLAL, the surface titanium atoms are removed when the laser fluence is more than the ablation threshold of titanium. The removed material forms a plasma plume. The optical breakdown of the target occurs due to irradiation by laser pulse to form plasma directly from solid [8]. The cavitation bubbles formed as a result of expansion of plasma consist of not only ablated species from the target but also solvent molecules. Lam et al. studied the bubble expansion following PLAL and reported the presence of mostly solvent molecules in the cavitation bubble [23]. The extreme conditions of the plasma plume and cavitation bubbles provide a thermodynamic window in which metastable phases can also be formed along with the stable phases; however, phase transformation is possible as the process proceeds. For PLAL in DIW, the formation of hydrated products is possible when unusual focusing configurations are used, such as when the target is not at focus but just under focus so that the laser causes breakdown of water just above it [14]. However, in our experiments, the laser was focused on the target surface. Other phases can form also, but as mentioned earlier, further transformations can happen as the process continues. The ablation of titanium target dipped under 5-mm deionized water was observed to begin at a laser fluence of ~13.85 J/cm^2^.

The experiments were conducted with the pulsed laser at 25-kHz repetition rate, which means the delay between two laser pulses was 40 μs. This is much shorter than the time duration of a cavitation bubble which is usually ~300–680 μs depending on laser parameters and solvent properties. This results in strong screening of the laser for every pulse after the first pulse. The fully expanded cavitation bubble acts as a negative lens and strongly defocusses the incident laser beam, thereby reducing the laser fluence at the target surface. This strong defocusing of subsequent laser pulses by bubble formed by previous pulse causes screening of laser pulses and results in irregular ablation by the successive pulses [22]. With the laser and scanning parameters used (25 kHz repetition rate, 500 ns pulse duration, 2000 mm/s scanning speed, and 8 × 8 mm scanning area), the distance between two laser pulses on the target surface was 80 μm. This is smaller than the spot size of the laser, especially after the defocusing caused by cavitation bubbles. Due to this, re-ablation on the same spot occurs which raises the temperature of the plasma formed by previous laser pulse. Moreover, in PLAL using a nanosecond laser, there is a temporal overlap between the laser pulses and the ablated material. The plasma plume formed by the laser pulse absorbs part of the energy from the subsequent laser pulse and results in optical shielding of the target from the laser pulses [14]. This also increases the temperature of the plasma plume and decreases in the energy absorbed by the target surface. As the PLAL process continues, more and more nanoparticles are released in the surrounding solvent which interact with the incident laser pulses and absorb its energy. The laser beam in the experiments is focused at the target, so the synthesized nanoparticles undergo laser processing without laser beam focusing on them. For this configuration with a nanosecond laser, Pyantenko et al. reported that the nanoparticles get intensely heated by the energy absorbed from the laser pulses and can evaporate or explode to form smaller fragments, thereby causing size reduction [24]. Werner et al. concluded that the photothermal mechanism alone (without Coulomb explosion) can explain this pulsed laser-induced size reduction of nanoparticles with nanosecond pulsed laser [25].

### Characterization of Nanoparticles by TEM and XRD

**Fig. 2:**
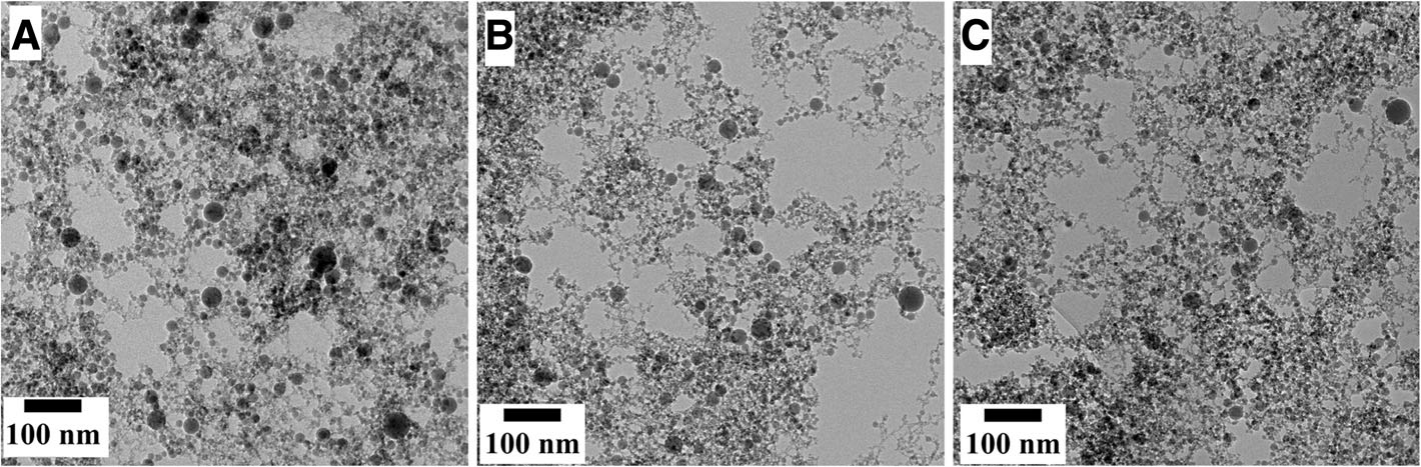
TEM images show round nanoparticles synthesized at (**a**) 20 %, (**b**) 40 %, and (**c**) 50 % LP

The synthesized suspensions were found to contain spherical nanoparticles when analyzed with TEM. In Fig. 2a–c, the TEM images show the nanoparticles synthesized at 20 % LP, 40 % LP, and 50 % LP were all almost perfectly round. The nanoparticles assume spherical shape in order to minimize the interfacial energy. The nuclei consisting of cluster of atoms coalesce with other nuclei to form polycrystalline nanoparticles [14]. The presence of almost perfectly round nanoparticles implies that when nuclei coalesce, either they are melted or their temperature is high enough to ensure the mobility of surface atoms [20]. However, the aggregate of nanoparticles can also undergo melting, fusing, and merging to form larger spherical nanoparticles if the laser fluence is high enough [24]. The nanoparticle suspension synthesized by 20 % LP, Fig. 2a, consisted of nanoparticles whose diameters varied from ~4 to ~35 nm with about 80 % of the nanoparticles less than 14 nm in diameter.

**Fig. 3:**
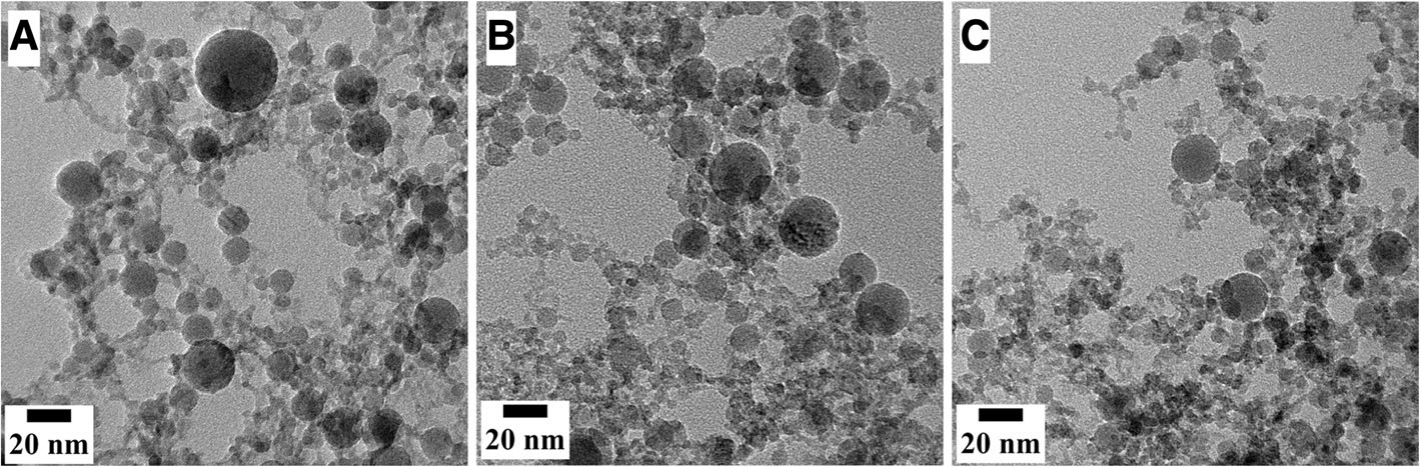
TEM images show nanoparticles forming web-like structure (**a**) 20 %, (**b**) 40 %, and (**c**) 50 % LP suspensions

The TEM images in Fig. 3 show web-like structure of nanoparticles synthesized at (a) 20 % LP, (b) 40 % LP, and (c) 50 % LP. At this contrast, the particles can be easily distinguished. Ledoux et al. also reported this web-like structure of nanoparticles formed as a result of PLAL [26]. Also, evident from Fig. 3 is the decrease in size of particles from panel a to b to finally c. The scale bar is 20 nm, and the dimension of scale bars in each image is the same. Another important observation from Fig. 3 is the presence of mostly small nanoparticles; however, relatively larger nanoparticles were also present. This indicates a bimodal size distribution for the synthesized nanoparticles. Such bimodal size distributions of nanoparticles produced by PLAL were also reported by Meunier and Meneghetti [14, 27]. Further analysis of these two observations, (i) decrease in mean size and (ii) size distribution, is covered in the “Particle size analysis” section.

**Fig. 4:**
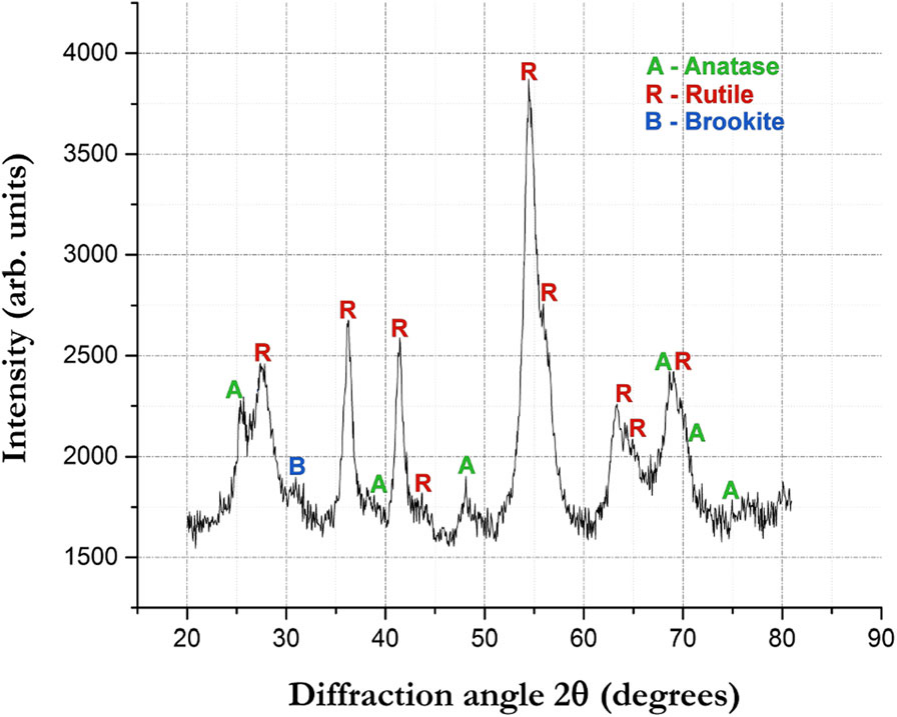
XRD pattern showed anatase, rutile, and brookite in 40 % LP nanoparticle suspension

Electron diffraction patterns from TEM samples of all three suspensions indicated mainly crystalline particles which agrees with XRD results (Fig. 4) as high and sharp peaks were visible in XRD pattern indicating high crystallinity. Phase identification with XRD showed that the particles synthesized by PLAL of titanium were TiO_2_ nanoparticles. They were present in the form of multiphase titania, consisting of photoactive allotropes, mostly anatase and rutile. Due to small size of particles, broadening of the peaks occurred. The most important peaks for anatase (2*θ* = 25.4), and rutile (2*θ* = 27.4), were detected along with many other peaks corresponding to them in the XRD pattern as shown in Fig. 4. The small peak at 2*θ* = 30.8, which is the highest intensity peak for brookite, indicated its presence. As the peak is quite weak, it signifies presence of extremely small amount of brookite. Rutile was the major phase of the nanoparticles while anatase and, in particular, brookite were the minor phases. These X-ray diffraction patterns were very similar for all three suspensions, and the ratio of TiO_2_ phases was also the same indicating that the amount of titania phases synthesized does not vary with the change in laser power for the laser and scanning parameters used. In addition, as anticipated, there were no titanium peaks in the pattern which signifies that all the atoms and clusters of titanium, ablated or droplets ejected from the target, interacted with the solution species. This is understandable considering titanium is chemically active metal, and unlike noble metals, it should not form pure metal nanoparticles.

### Particle Size Analysis

Manual measurement of the nanoparticle size from the TEM images gives a number distribution. It is a time-consuming method, and the total number of particles examined is a very small fraction of the total number of nanoparticles. So, it can be argued whether the TEM sample is representative of the bulk of the suspension. In contrast, SAXS gives volume size distribution and corresponding mean diameter by measurements from much more nanoparticles compared to TEM. The SAXS measurement follows a parametric distribution model method. In this distribution model method, the particle shape is assumed to be the same, spherical in this case, while the size of the particle varies [15].

**Fig. 5:**
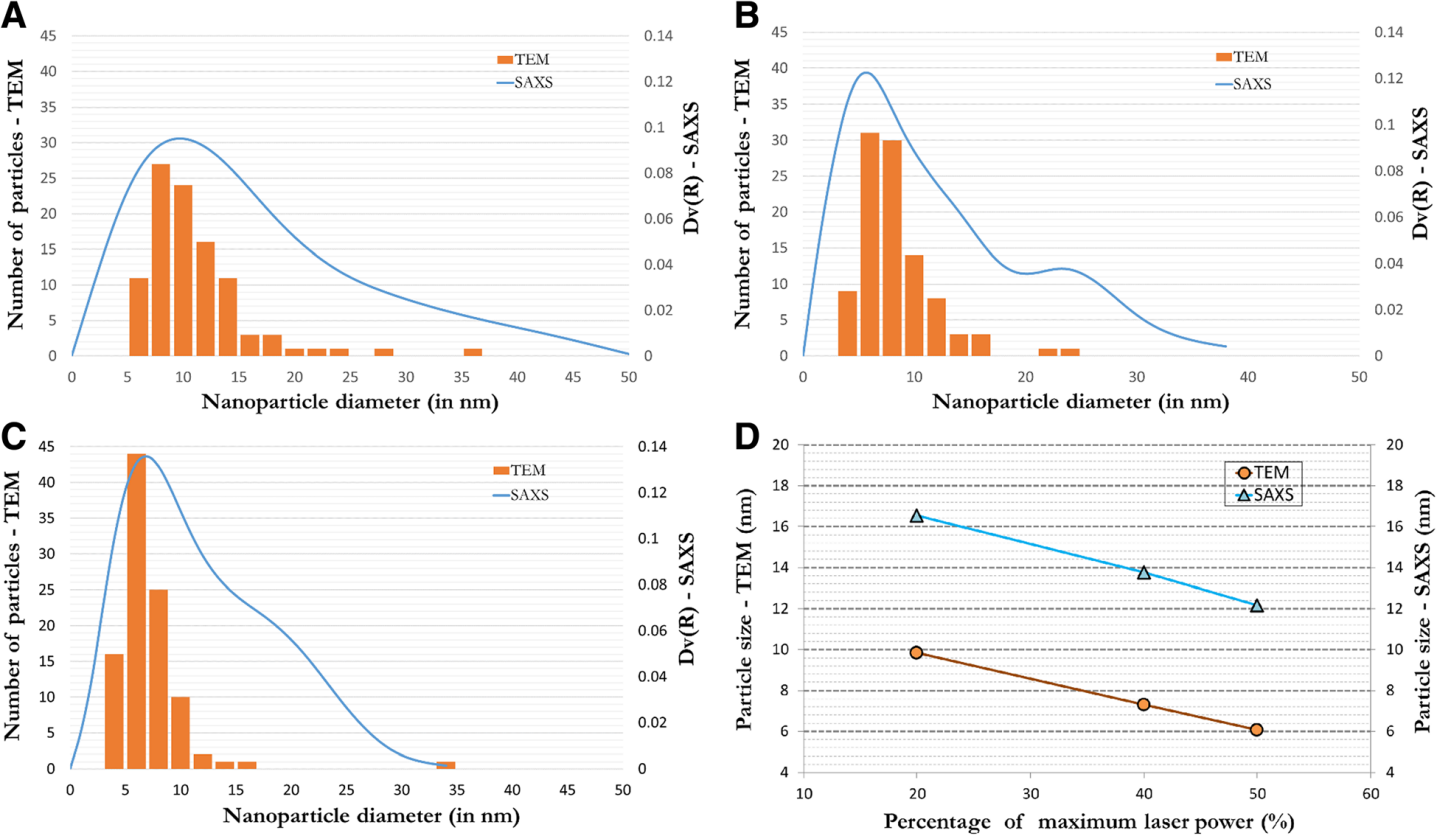
Particle size distributions. **a** 20 %, **b** 40 %, and **c** 50 % LP suspensions. **d** TEM versus SAXS average diameters

In order to compare the two techniques, TEM and SAXS, the size distributions were plotted on the same plot. Figure 5a–c shows the histogram of nanoparticle diameter versus the number of particles on the left *y*-axis, which was determined from TEM images manually, along with Dv(R) from SAXS on the right *y*-axis versus the same nanoparticle diameter on the *x*-axis. The particle size distribution information from the histograms (obtained from TEM) is appropriately represented by the size distribution curve (bimodal size distribution obtained from SAXS). In each case, the curve seems to be following the histogram very well. In Fig. 5a, the size distribution curve and histogram follow each other well. The histogram shows the maxima at 8–10-nm nanoparticle diameter which is extremely close to the maxima of SAXS-measured size distribution curve at 10-nm nanoparticle diameter. This is followed by a drop in the number of nanoparticles for both measurements as the nanoparticle diameter increases. In Fig. 5b, the maximum of the size distribution curve lies yet again extremely close to the maxima of histogram. The histogram and size distribution curve both show the presence of increased number of nanoparticles with diameter 22–24 nm after a minimum at around 19-nm diameter. In Fig. 5c, the maxima of both histogram and size distribution coincide at around 6-nm nanoparticle diameter. Another important observation was the size distribution, determined by both techniques, became narrower with the increase in the laser power from 20 to 40 % to finally 50 %. This is due to photothermal melting-evaporation and explosive fragmentation of larger nanoparticles to form smaller nanoparticles which narrowed the size distribution [24, 25]. These mechanisms for interaction between laser pulses and target materials change if the pulse duration of the laser is shortened, such as in femtosecond and picosecond lasers, the mechanism changes to Coulomb explosion. Werner et al. have established a thermodynamic model to interpret the effect of laser parameters on the size reduction mechanisms for nanoparticles [25]. The results from both techniques, SAXS and TEM, are consistent with each other for the measurement of TiO_2_ nanoparticle size distribution.

The mean nanoparticle diameters showed in Fig. 5a–c were 9.84, 7.31, and 6.06 nm, respectively, from TEM images, while for the same samples analyzed by SAXS measurement, the mean nanoparticle diameters were 16.54, 13.76, and 12.16 nm, respectively. The measured mean nanoparticle diameters in SAXS technique and from analysis of TEM images varied from each other for the same samples. The nanoparticle diameters measured by TEM image analysis were smaller compared to the respective nanoparticle diameters measured by SAXS measurement. The crystallite sizes were measured from XRD pattern of the dried 40 % LP suspension (Fig. 4). The Scherrer results from XRD pattern using Panalytical’s HighScore Plus software (version 3.0.5) showed that crystallite sizes were in the range 3.9 to 13.3 nm with a mean crystallite size of 8.01 nm. For the same sample, the mean diameter of TiO_2_ nanoparticle measured by TEM and SAXS was 7.31 and 13.76 nm. The TEM-measured particle size was in agreement with the crystallite sizes measured by XRD. However, the SAXS-measured nanoparticle diameters were slightly higher than TEM-measured diameters for each sample. There are some important reasons for this slight deviation from TEM-measured nanoparticle diameters.

Firstly, while TEM image analysis gives a number size distribution, the SAXS measurement gives volume size distribution. So, in SAXS measurement, the mean volume of sphere is measured which is then converted into the corresponding mean diameter of nanoparticle. Thus, the principle of determination of mean diameter for both measurement techniques is different. This can be explained by assuming three spherical nanoparticles with diameters 2, 6, and 10 nm. According to TEM image analysis with a number distribution, the mean particle diameter is 6 nm while with SAXS measurement, which is volume distribution, the mean particle diameter is 7.42 nm (the three spherical nanoparticles with diameters 2, 6, and 10 nm correspond to volumes of 4.18, 113.04, and 523.33 nm^3^, respectively. This gives an average volume of 213.52 nm^3^, from which the SAXS-deduced average diameter is 7.42 nm).

Secondly, it is well known that in XRD, the diffraction occurs at the periphery of the crystallite (for bulk samples) and through the volume (for thin samples such as nanoparticles) while in X-ray scattering, elastic scattering of the X-rays occurs at the phase boundary. Due to this, while measuring with SAXS, several primary particles that are joint in the form of an agglomerate cannot be distinguished, and therefore, we obtain the size of agglomerate and not the primary particle. Due to this, SAXS measures an agglomerate as one big particle which gives a higher volume and consequently increases the mean nanoparticle diameter in the volume size distribution measurement. However, with TEM, we can see and distinguish the agglomerates from primary particles. The measured mean diameter from SAXS, 13.76 nm, is still very close to the mean particle size measured by TEM, which indicates that most particles were present as primary particles along with the presence of a small number of agglomerates containing few particles. Furthermore, from the SAXS measurement of the same sample, the most frequent diameter of the nanoparticles in 40 % LP suspension (Fig. 5b) was 7 nm. This is in agreement with the aforementioned hypothesis regarding presence of mostly primary particles along with a small number of agglomerates. This is also consistent with the TEM- and XRD-measured sizes, 7.31 and 8.01 nm, respectively. In addition, this is evident also from the Fig. 5b showing TEM histogram and SAXS-measured size distribution, both of which have maxima at the same particle size. Furthermore, if we see the trend of change in mean nanoparticle diameter from Fig. 5d, it is exactly the same for both measurement techniques indicating good agreement between the results from both techniques. The similarities in the plotted curves and the trend evidence the universality of the established SAXS measurement technique for the particle size and size distribution analysis of TiO_2_ nanoparticles.

Finally, it is important to mention that the SAXS was sensitive to the concentration of the nanoparticle suspension. In several samples, the absorption factor was not in the range 1.5–5. According to Panalytical’s guidelines for EasySAXS software, the results are then not reliable. Absorption factor less than 1.5 indicated that concentration of particles was so low that there was no conclusive difference between the nanoparticles and the background in SAXS measurement. For the same reasons, the suspensions with low particle concentration rendered so few amount of powder that it was not XRD detectable. With such small amounts of nanoparticle powders, specific surface area analysis by gas adsorption (BET) was also out of question. All these samples were obtained as a result of 30 min of ablation time. In order to increase the concentration, the ablation time can be increased. However, this was a batch process, and higher ablation times lead to increased laser processing of the already synthesized nanoparticles and result in stability issues with the suspension as the temperature of the liquid rises. Lam et al. limited the irradiation time to 20 min in order to keep the thermodynamic conditions stable throughout the experiment [28]. Although we propose SAXS for size and size distribution measurement of these nanoparticles, we still propose the use of TEM for determination of shape and appearance of nanoparticles, as we do not obtain this information from SAXS.

## Conclusions

Pulsed laser ablation in deionized water was demonstrated to be a promising method to synthesize TiO_2_ nanoparticles from titanium target. With 500-ns laser at 25 kHz scanning an area of 8 × 8 mm at a speed of 2000 mm/s, we determined that there was (i) temporal overlap between the laser pulses and the ablated material, (ii) strong screening of laser pulses by cavitation bubbles, and (iii) re-ablation. TEM results showed that the nanoparticles synthesized were spherical in shape, ranging in size from 4 to 35 nm with 80 % of them with diameters smaller than 14 nm for the suspension synthesized at 20 % laser power. XRD results indicated that the nanoparticles constituted mainly two allotropes of titania: anatase and rutile. The weak peak for brookite indicated its presence as a minor phase. Rutile was found to be the major phase of the synthesized nanoparticles.

For size distribution measurement of these nanoparticles, SAXS was introduced and evaluated with promising results. The SAXS-measured particle size distribution measurements were consistent with TEM. Both showed that the size distribution of nanoparticles became narrower with the increase in laser power from 20 to 40 % to finally 50 % while at the same time, the nanoparticles also became finer. This was attributed to explosive fragmentation of larger nanoparticles to form smaller nanoparticles and photothermal melting-evaporation of all nanoparticles during irradiation. Due to the difference in measurement type and sensitivity towards agglomerates, SAXS-measured diameters were slightly higher than TEM-measured diameters. However, the trend in the variation in the mean nanoparticle size with laser power determined for 20 % LP, 40 % LP, and 50 % LP suspensions by both techniques, SAXS and TEM, was exactly the same, which fortifies the discussion and the consistency in the results from both techniques. Based on the well-founded discussion, the SAXS-measured nanoparticle size and size distribution results are evidently propitious, noble, and promising. We propose SAXS as a promising method to measure particle size and size distribution of PLAL-synthesized TiO_2_ nanoparticles. However, in order to obtain comprehensive information on the shape and appearance of nanoparticles, we propose using TEM.

## Abbreviations

AFM: Atomic force microscopy
DLS: Dynamic light scattering
DMA: Differential mobility analysis
D_v_(R): Distribution volume ratio
LP: Laser power
PLAL: Pulsed laser ablation in liquids
SAXS: Small angle X-ray scattering
SEM: Scanning electron microscopy
TEM: Transmission electron microscopy
XRD: X-ray diffraction
